# EU Member States’ Institutional Twitter Campaigns on COVID-19 Vaccination: Analyses of Germany, Spain, France and Italy

**DOI:** 10.3390/vaccines11030619

**Published:** 2023-03-09

**Authors:** Jorge Tuñón Navarro, Emma Oporto Santofimia

**Affiliations:** Department of Communication, Carlos III University of Madrid, Calle Madrid 126, 28903 Getafe, Madrid, Spain

**Keywords:** European Union, digital strategic communication, social networks, Twitter campaign, COVID-19, vaccination

## Abstract

The development of an effective vaccine against the SARS-CoV-2 coronavirus became the hope for halting the spread of the disease. In recent years, social networks have become important tools for political and strategic communication in the dialogue with citizens. Therefore, the messages emitted through them were important to address vaccine hesitancy and achieve collective immunity. This paper analyses the use of Twitter by politicians and institutions in EU Member States during the first fifty days after the Commission’s marketing authorisation of the first COVID-19 vaccine (21 December 2020 to 8 February 2021). To do so, a triple approach content analysis was carried out (quantitative, qualitative and discursive on feelings) applied to 1913 tweets published by the official profiles of the prime ministers, health ministers, governments and health ministries of Germany, Spain, France and Italy, the four most populous EU countries. The results point out that politicians and institutions gave preference to other issues on their political agenda over vaccine-related issues. Moreover, previous research hypotheses, such as those related to the underutilization of the Twitter tool as a two-way communication channel with citizens, are validated.

## 1. Introduction

The pandemic caused by the SARS-CoV-2 coronavirus, which emerged during December 2019, has had significant social, health and economic consequences in the European Union (EU). In response, the scientific community developed an effective and safe vaccine in record time as a hope for achieving herd immunity and halting the spread of COVID-19.

Although vaccines have become the most important public health measure for the control and eradication of infectious diseases [1], societal controversy surrounding vaccines has existed since the development of the first smallpox vaccine [2]. Recently, anti-vaccine movements have reappeared. Their messages may increase public mistrust regarding vaccination safety. In this regard, the World Health Organization (WHO) has considered reluctance or refusal to vaccinate as one of the greatest threats to global health [3].

Anti-vaccination groups have found the internet and social media to be eco chambers in which to convey their messages and, therefore, encourage misinformation about vaccines [4,5]. Indeed, institutions and political stakeholders should enjoy a digital presence, because their communications about vaccination campaigns can contribute to addressing vaccine hesitancy—by disseminating verified information—and to achieving herd immunity. Among social media, “Twitter may represent a powerful public health tool for world leaders to rapidly and directly communicate information on COVID-19 to citizens, in addition to more conventional media such as television, radio and newspapers” [6].

Accordingly, this paper aims to investigate the use of Twitter by politicians and institutions in four EU Member States, Germany, Spain, France and Italy, during the vaccination campaign against COVID-19, precisely as a measure against sceptical or populist waves of disinformation on the dissemination of vaccination among European citizens.

### 1.1. Political and Institutional Communication 2.0

Since its emergence, the Internet has become an essential element in practically all areas that make up the daily lives of individuals. It has given rise to the so-called “Network Society”. Castells [7] defines it as a global society whose social structure is built around digital networks. As indicated in the annual report Digital 2021: Global Digital Overview, produced by We are Social and Hootsuite, 4.66 billion inhabitants use the Internet, i.e., 59.5% of the world’s population [8].

The emergence of the so-called Web 2.0 [9] transformed the way people communicate with each other. The consolidation of social networks led to a shift from a passive user who only consumed messages to a more active user who interacts, collaborates and generates content. The horizontal model of communication offered by social media allows citizens, politicians and institutions to coexist on equal terms by breaking the classic paradigm of sender–message–receiver communication, with its corresponding exchange of roles between actors.

Previously, politicians and institutions transmitted their messages through the media and the audience received them almost without the option of intervening. Now, both can disseminate their information without using the mass media as intermediaries [10]. Moreover, citizens can debate with them, and be simultaneously transmitters and receivers of information [11]. Consequently, political stakeholders and public institutions have been using social media as relevant instruments in their communication strategies and in the dialogue with citizens [12]. 

However, scholars warn that both fail to exploit their potential as two-way communication channels [13,14]. Although they include its use in their communication strategy, they do so from a unidirectional communication, typical of conventional media (press, radio, television). For Calvo [15], although they are present in these spaces, most of them are still anchored in the former mass communication models, without understanding that the message is the people. Mancera and Pano [16] agree when they point out that the question is not simply whether or not to have an official profile, but to participate actively.

Accordingly, Campos [17] highlights that, instead of encouraging the debate with users, managing their comments and, therefore, establishing a two-way conversation, politicians seek exclusively to ensure that their messages reach the largest audience, establishing themselves, then, as a mere “noticeboard” for their actions and activities.

### 1.2. Digital Communication on Twitter

In this context, although Facebook continues to be the online network with the largest community of registered users on a global scale, Twitter is the preponderant social network by its very nature both for the debate and implementation of communication strategies of political organizations and for scientific studies in the area of Social Sciences in this specific field [18,19,20]. Having made the above explicit, this does not imply that we are advocating that one network is better for information dissemination, but rather that we should delve deeper into the fact that each of them is oriented to a different audience and/or demographic group. Created by Jack Dorsey in 2006, the microblogging network Twitter made it possible to publish messages of any type in a maximum of 140 (now 280) characters in a public, immediate and easy way [21]. It has become an effective platform by providing a space in which citizens, politicians and institutions can communicate without intermediaries [22]. Moreover, it has functions such as hashtags, which allow messages to be disseminated on a large scale, along with a tendency to go viral. It also has retweets and ‘likes’, through which users can also spread content [22].

The change in political and institutional communication brought about by social media has recently led to the study of hybridization in the field of political and institutional communication, i.e., the symbiosis between the digital environment and traditional media [23]. Therefore, politicians and institutions enhance traditional media logics with the new media logics provided by social networks. Aware of the relevance of conventional media for communication, they continue to rely on it while at the same time encouraging the use of digital tools [24]. Indeed, Bouza and Tuñón [25] point out that interaction. The frequency with which Twitter is used peaks during the retransmission of political speeches through traditional media [26], at the same time as the microblogging network expands and makes the political speeches more visible on the agenda of traditional media [27].

In general, two uses of Twitter stand out in the fields of political and institutional communication: as a tool for disseminating their messages, as well as for mobilizing and inciting users to action [28]; and as a way of transmitting information that links their corporate website or other networks. With regard to the above, Caldevilla [29] considers it a mistake to measure the level of importance of a political stakeholder by the number of followers it has on social networks. On the contrary, its use as an alternative media is established as its greatest strength. 

The academic literature on the use of Twitter by political figures dates back to Barack Obama’s first presidential campaign in 2008. Since then, it has had three main focuses. The first focuses on citizens and politicians as senders and receivers. The second focuses on the political debate on social media and its effects. The last revolves around electoral campaigns, with special emphasis on its use and agenda [17].

### 1.3. Pandemic and Vaccination Institutional Strategies Management

Besides institutional communication, vaccination processes at a European level should also be pointed out. It has been noted that despite their proven effectiveness in saving lives and in containing and eradicating disease [30], vaccines have often remained surrounded by controversy [31] and have even, in many cases, faced outright opposition [32]. The reasons for this distrust derive, among others, from a postmodern cultural context that questions the legitimacy of science, the pharmaceutical industry and medical authority [33], and from a range of controversies about possible negative side effects associated with vaccines [34]. 

Doubt and suspicion have spread across the globe [35]. This has led the WHO to include mistrust of vaccines in its list of the top ten global health threats [36]. In this respect, the COVID-19 vaccination process has not been an exception. One of the main reasons for distrust among those who speak out against receiving the vaccine has been fear of possible side effects, far ahead of other possible causes, including the speed with which clinical trials have been conducted or other conspiracy theories such as that vaccines were designed to take control of human brains. 

Much of this dialogue about the advisability or otherwise of vaccines has shifted from conventional media to social networks [37,38], although not always with guarantees of reliability in the face of fake news [39]. Therefore, institutional campaigns at both national and European level (despite there being some common standards at EU level, such as the common centralized vaccines purchase or the partial periodic distribution among the EU members, member states settled down their own vaccination protocols) were launched as soon as the first vials patented began to be administered in all European territories. Against this backdrop (due to vaccination reluctance or European communication shortcomings at national level), this study addresses both the institutional management strategies of national governments and leaders and the digital organizational communication strategies implemented by the largest EU member states, through a tool now considered essential in the field of political and organizational communication: the social network Twitter (21). 

COVID-19 has attracted the attention of the academic literature since its inception, which has resulted in an increase in papers in scientific journals over time. In the field of social media, which is increasingly used to inform about public health measures, the main focus has been on what have been called the two pandemics: the coronavirus and the disinformation [40,41,42,43]. However, research has also been conducted from a political and institutional approach [44,45,46,47].

In this regard, Wu et al. [48] recommend that institutions consider three strategic principles in their response to the pandemic. First, to provide resilience-based leadership. Second, to articulate crisis communications to provide the most up-to-date data on the coronavirus and anticipate possible questions from citizens. Finally, continuous support to health workers.

In the specific case of vaccination, scholars have also addressed disinformation, with a focus on vaccine reluctance and anti-vaccine movements [49,50]. Despite the relevance of social media in political and institutional communication, the literature related to the communicative strategies played by both political actors and public institutions around vaccines is lacking. However, and closely related to the above, there are more works which with a more or less broad transnational focus have concentrated on the analysis of the acceptance of the COVID-19 vaccine [51] or the willingness to use it [52], also as a consequence of institutional campaigns to promote it.

As long as having a vaccine does not automatically imply it will be used, addressing the scope of COVID-19 vaccine hesitancy in various countries is recommended as an initial step for building trust in COVID-19 vaccination efforts. It is therefore important to understand whether or not people are willing to be vaccinated against COVID-19, as this may have important implications for the success of vaccination institutional strategies worldwide, with potentially significant health and economic consequences. Indeed, comparative previous analysis, conducted in up to three of our sample countries (France, Italy and Spain), has also pointed out that “effective coordination between governance levels, ability to ensure a large supply of doses, and trust towards health authorities were amongst the determinants for successful outcomes of vaccination campaigns” [53]. 

Among our chosen countries, Spain and Germany enjoy the highest COVID 19 vaccine acceptation and willingness to voluntarily be administered the doses. In the German case, up to 70% adults would voluntarily get vaccinated against the coronavirus if a vaccine without side effects was available [54]. The relationship between the exposure to and credibility of different health information sources and the COVID-19 vaccination intention has also been tested. Indeed, reliable information from experts and health authorities’ campaigns were highlighted as the main factors encouraging vaccination acceptance and rates in Germany. Therefore, a close cooperation between healthcare experts, health authorities, and mass media with regard to information dissemination is conducive for vaccination campaigns and for the fight against misleading claims about COVID-19 vaccines [55]. However, other studies in Germany detected considerable regional differences in the efficiency of the vaccination roll-out [56].

For its part, France has been exhibiting some of the lowest rates of uptake and interest in dosing throughout the vaccination period [53]. According to research conducted by Cambon et al. [57], the French government’s communication strategies have not helped; in fact, they have worsened the situation. In addition, the work notes that empirical studies on the national strategy for the management of the COVID-19 pandemic in France have shed light on the reasons for vaccine hesitancy. These studies have identified, among the four pillars of the failure of the vaccination strategy, the (institutional) communication about the importance of herd immunity. Guillon and Kergall [58] add about the French situation that awareness campaigns should be conducted and improved to enhance vaccination uptake among vaccine hesitant individuals.

In contrast to the Spanish case, Italy [59], like France, has had a higher level of resistance to the vaccine, as well as lower vaccination rates. Traditionally, this has been attributed to both cultural factors and resistance to the government in power. Therefore, works such as the one by Antonini et al. [53] argue that countries with higher levels of hesitancy could promote targeted campaigns to increase vaccine uptake among hesitant groups rather than relying (only) on boosters as a mechanism to reduce the impact on the health system and the economy.

Notwithstanding the above, for the Italian case other recent research such as Bucchi et al. [60] points to positive evaluations of experts’ communication and trust in their contribution—as well as that of health institutions, local authorities and health workers—as playing a key role in understanding willingness to vaccinate. Therefore, they add that relevant implications can be drawn in terms of communication efforts and institutional strategies that are essential for building effective and inclusive vaccination campaigns.

Although it is outside the geographical context analysed in this research, it is necessary to make reference to the online institutional communication about vaccination in the context of the pandemic, and to the work of Benis et al. [61], which analyses intentions towards vaccination versus COVID-19 on networks, including Twitter, and highlights the importance of governments including social media as part of their communication strategy to convey information about vaccines, build trust in vaccines and thus increase vaccination rates. Similarly, recent research by Herrera et al. [62] is worth mentioning. The study analyses a sample of tweets in Spanish from the hashtag #yomevacuno published between 8 December 2020 and 23 December, 2020. Five of the most influential users belonged to official profiles of the European Union (European Medicines Agency—Amsterdam, The Netherlands and of the Spanish government (Ministry of Health and La Moncloa). According to the research, institutions should lead public health communication through social media by providing verified information.

Given the above, our research humbly aims to contribute to filling the existing academic gap by studying EU members’ institutional digital Twitter campaigns on COVID-19 vaccination in Germany, Spain, France and Italy.

## 2. Materials and Methods

### 2.1. Objectives and Hypotheses

The aim of this investigation is to analyse how the social network Twitter was used by politicians and institutions in EU Member States during the COVID-19 vaccination campaign. For this purpose, four case studies have been selected: Germany, Spain, France and Italy. The limitation of the scope of the research to these territories is justified because they are the four most populated countries in the EU, and the most affected by the pandemic in the period under study.

In order to achieve the general objective, four specific objectives were suggested:

**O1.** Observe the level of interaction (one-way or two-way) between prime ministers, health ministers, governments and health ministries and users on Twitter, measuring the degree of commitment or engagement through retweets, likes and comments.

**O2.** Study the use that these politicians and institutions make of Twitter’s discursive resources (images, videos, links, etc.).

**O3.** Identify whether vaccination constituted the thematic agenda raised by the official profiles of the selected politicians and institutions and, if so, assess to what extent.

**O4.** Examine the sentiments attached to the vaccine messages of these politicians and institutions.

Based on these objectives, the following four working hypotheses were formulated, to seek to be tested and validated in the course of our research:

**H1.** In the digital interactions (about vaccination) of politicians and institutions with citizens, uni-directionality prevails over bi-directionality. Therefore, politicians and institutions consolidate the use of Twitter as part of their vaccination communication strategy. Both participate actively and exploit the resources offered by the tool, such as the inclusion of images or links that redirect to the corporate website for further information.

**H2.** The main content of the messages focuses on issues related to the political and institutional agenda, leaving vaccine-related issues in the background.

**H3.** The messages published by the European institutions and leading European officials do not present an accentuated positive polarity of a hopeful nature.

**H4.** The contents of publications related to the vaccine tend to generate more engagement than those that differ from the vaccine.

In some ways, the presentation of different hypotheses could be considered as the major findings of multiple case studies on communication strategy and its effectiveness in implementing public health policy. Even if the institutional bodies and the leaders communicate nationally, the world pandemic issue and the EU common health policies at stake make it inevitable to understand that their communication strategy is oriented towards pan-European and international audiences as well. 

### 2.2. Methodology

In order to achieve the research objectives, a methodological triangulation was designed to compare the four case studies (Germany, Spain, France and Italy). On the one hand, a quantitative analysis of the use of Twitter in the official profiles was carried out. For this purpose, the following variables were examined: 1. Number of followers at the time of data extraction. 2. Number of profiles followed. 3. Number of tweets. 4. Number of retweets obtained by each tweet. 5. Number of ‘likes’. 6. Number of comments. These last three were designed to determine the degree of user engagement.

On the other hand, a qualitative analysis (content and sentiment analysis) of the posts was developed. The former studies the content of messages in order to classify and/or code them into categories. The second seeks to determine the emotional tone of posts and categorize them as positive, negative or neutral [63]. On the one hand, content analysis is one of the most widely used research techniques in the social sciences, and makes it possible to understand the manifest and latent content of texts, as well as the codes used by their issuer and the context in which they arise and develop [64]. Therefore, unlike other methods it combines observation and data production with the interpretation or study of the data. On the other hand, sentiment analysis is a technique used in different fields that allows for a better understanding of the role played by emotions in social media systems, which are commonly used in these areas [65].

In order to carry out the research, both the accounts of politicians and those of the state institutions responsible for the management of vaccination were selected. To ensure homogeneity, the profiles of prime ministers, health ministers, governments and health ministries of the four countries were considered. 

All in all, a triple approach content analysis was carried out (quantitative, qualitative and discursive on feelings), already tested in previous research [66,67,68,69] applied to 1913 tweets published by the official profiles of the prime ministers, health ministers, governments and health ministries of Germany, Spain, France and Italy, the four most populous EU countries.

Therefore, the initial sample focused on Angela Merkel (German Chancellor), Pedro Sánchez (Prime Minister of Spain), Emmanuel Macron (President of the French Republic), Giuseppe Conte (Italian Prime Minister), Jens Spahn (German Health Minister), Salvador Illa (Spanish Health Minister), Olivier Véran (French Health Minister), Roberto Speranza (Italian Health Minister), the accounts of the four governments and those of the four Ministries of Health.

However, a number of adjustments had to be made to the sample chosen. Merkel had to be excluded (because she does not have an official profile on the social network) and replaced by Steffen Seibert, the then-spokesperson for the Federal Government and the German Chancellor. The German government was also excluded from the investigation due to the lack of use of the tool.

Next, the time frame of analysis was delimited. As the aim of the research was to analyse how politicians and institutions in EU Member States use Twitter during the COVID-19 vaccination campaign, the sample covers 50 days from the Commission’s marketing authorization of the first COVID-19 vaccine (21 December 2020 to 8 February 2021). This period selected covers the milestones of the three initial vaccines licensed for use in the EU. The BioNTech-Pfizer was approved on 21 December 2020, the Moderna vaccine on 6 January 2021 and the AstraZeneca vaccine on 29 January 2021. In addition, a few days were kept for the latter to observe its potential impact. 

It should also be noted that on 26 January 2021 Salvador Illa left the Spanish Ministry of Health and was replaced by Carolina Darias. Thus, both profiles had to be considered when the changeover took place within the chosen framework.

The tweets were then obtained. Initially, the data from the accounts that make up the sample were going to be extracted through the Twitter researcher API (Application Programming Interface) and managed with programs used in previous work, such as Gephi or Tweet Catcher by Chorus. However, these have not yet been updated to the new API, making it impossible to download tweets and retweets.

Consequently, we opted to use the microblogging network’s ‘Advanced Search’, manually acquiring all the messages from each institutional and political account. As this system prevents us from retrieving retweets, we considered instead the tweets quoted or mentioned which, although they are different concepts, can be used to replace retweets in hypotheses of the uni-directionality and bi-directionality of the posts. The study therefore only considers the tweets themselves.

Finally, for the qualitative analysis, a model form was developed (see Appendix A and Appendix B) after an initial screening of 10% of the sample, following some similar previous research models [27,70,71]. Four variables were considered: ‘Type’ (with three categories), ‘Format’ (with six categories), ‘Content’ (with two categories, with seven and nine subcategories, respectively) and ‘Polarity’ (with three categories, with six and two subcategories, respectively).

It should be noted that these are non-excludable categories. Therefore, messages could be classified in more than one category at a time. For example, when an institution or politician posted a tweet with an image and a link it was categorized as ‘Image’ and as ‘Link to external website/other networks’.

To create the engagement index, the system suggested by Ballesteros [72] was followed, which consists of finding ten percentiles of the variables ‘Number of retweets’, ‘Number of likes’ and ‘Number of comments’ to prorate the sample and obtain homogeneous groups for the analysis (see Appendix B). This decision arose after finding deviations before the start of the analysis. In this line, publications with a number of ‘likes’ within the first decile were categorized with a 1, within the second decile with a 2, and so on. The SPSS Statistics V21 program was used to work with the data, which allowed us to fill in the data sheet models presented above and to interpret the results presented in the next section.

Therefore, the corpus of analysis is made up of 1913 tweets, which are divided among the sixteen politicians and institutions that finally formed the sample of Twitter profiles analysed (see Table 1).

## 3. Results

### 3.1. Case Study: Germany

The government spokesperson, the Minister of Health and the Ministry of Health use Twitter via the accounts @RegSprecher, @jensspahn and @BMG_Bund. At the time of data extraction (5 June 2021), Seibert led in number of tweets, number of followers and in followers/followed profiles (see Table 2). All three issued 355 posts during the study period, 18.6% of the total.

Regarding the type of messages, 95.2% are tweets while the remaining 4.8% are mentions (2.8%) and replies (2%). Only Spahn exceeded 3% of mentions. Regarding replies, Seibert replied to 2.9% of his posts, the BMG to 2% and Spahn to none. Consequently, all have a low level of interaction.

**Figure 1:**
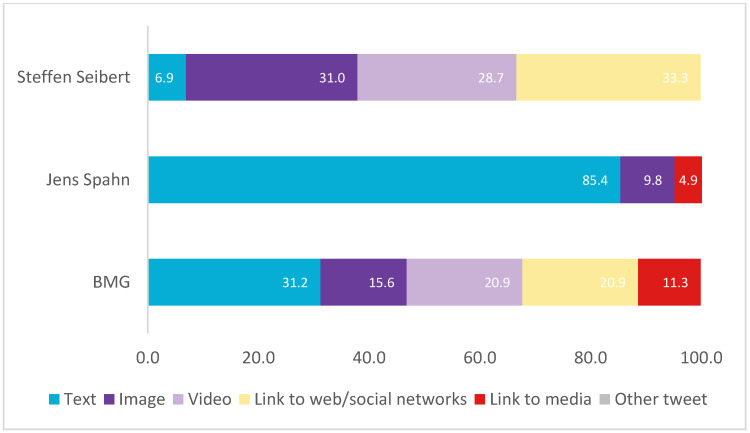
Typology of message formats in Germany as a percentage of the total number of messages. Source: Twitter and own elaboration.

In terms of format, Seibert accompanies more than half of his posts (59.7%) with images (image and video), while the Ministry and Spahn make more limited use (36.5% and 9.8%, respectively) (see Figure 1).

**Figure 2:**
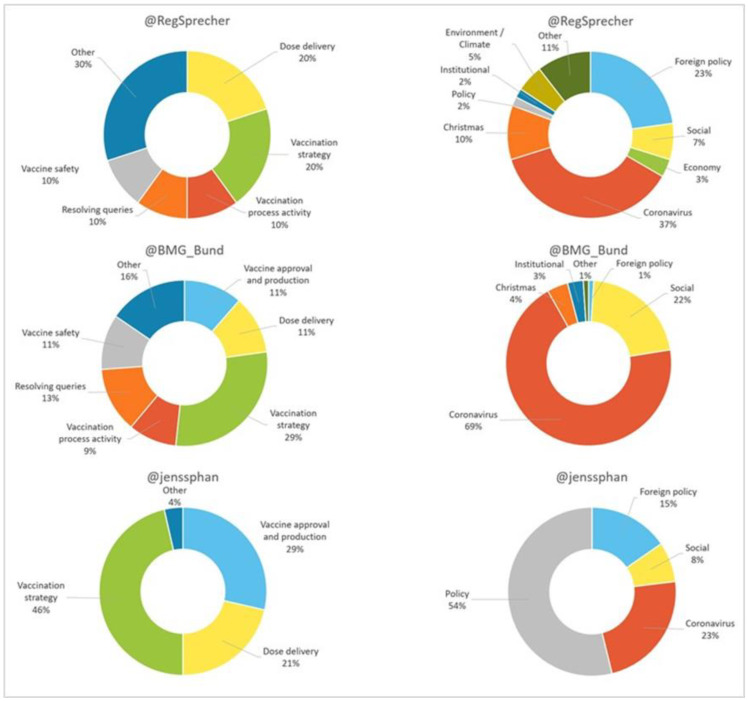
Content (vaccine related/other) of digital messages in Germany as a percentage. Source: Twitter and own elaboration.

Regarding the content (see Figure 2), most of Spahn’s (68.3%) and BMG’s (60.3%) tweets talk about the vaccine. Seibert is the exception, as 85.1% of his tweets do not refer to the vaccine. Within the vaccine-related topics, “vaccination strategy” predominates in the profiles of the Minister and the Ministry (46% and 29%, respectively) while for the Spokesperson it is the category “other” (30%). For content other than vaccination, “coronavirus” dominates Seibert’s and the BMG’s profiles (37% and 69%, respectively) and “politics” Spahn’s (54%).

**Figure 3:**
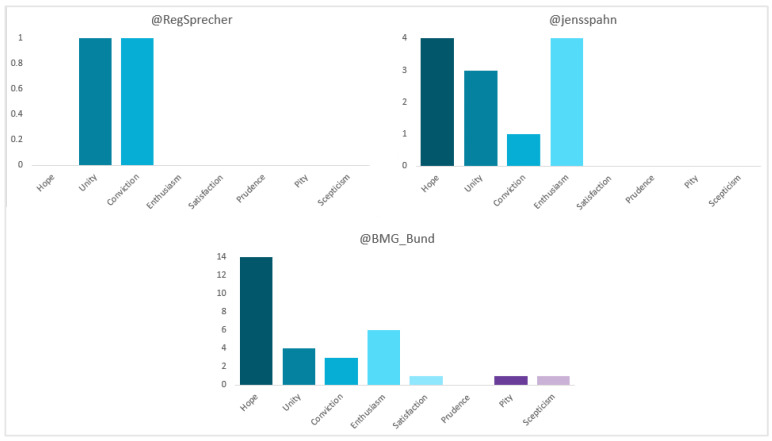
Sentiment of the tweets of German politicians and institutions. Source: Twitter and own elaboration.

As far as polarity is concerned (see Figure 3), 80% of Seibert’s posts are “neutral” and 20% “positive”. “Unity” (50%) and “conviction” (50%) are the only sentiments he appeals to. Spahn’s are more balanced (57.1% and 42.9%, respectively). On the other hand, “hope” and “enthusiasm” are the most used, with 33.3% each.

The Ministry is the only one to present all three types of polarity, with a majority (79.9%) “neutral”, 18.8% “positive” and 1.3% “negative”. Hope” is the predominant positive sentiment (50%). Of the two negative publications, one radiates “pity” at Pfizer’s failure to deliver the promised volume of doses, while the other conveys “scepticism” at the high number of new infections despite the start of vaccination.

**Figure 4:**
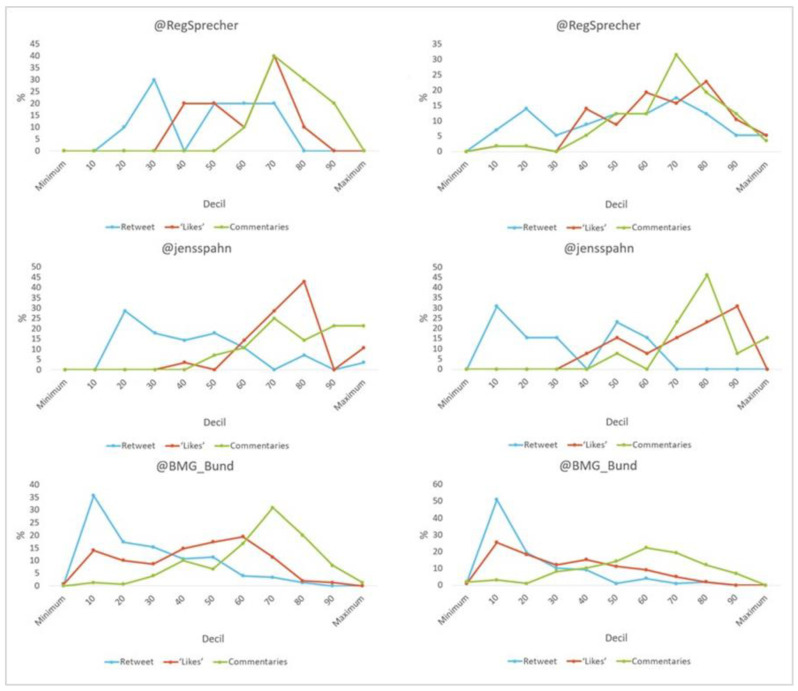
Engagement as a percentage of tweet content (total/vaccine-related) by decile in Germany. Source: Twitter and own elaboration.

With regard to the level of engagement (see Figure 4), it is noteworthy that all (100%) Seibert’s tweets about the vaccine, 89.3% of Spahn’s and 98.7% of the BMG’s do not exceed 100 “retweets”. However, half (50%) of Seibert’s posts do so in number of “comments”. With regard to content other than vaccination, more than half (53.9%) of Spahn’s posts exceed 100 “likes”. Furthermore, 51% of the Ministry’s retweets are in decile 1, i.e., between 10 and 18 times shared. Likewise, the latter remains with messages with no interaction on both vaccine-related and non-vaccine-related issues. 

### 3.2. Case Study: Spain

The Prime Minister, the Minister of Health, the Ministry of Health and the Government use Twitter with the accounts @sanchezcastejon, @salvadorilla, @CarolinaDarias, @desdelamoncloa and @sanidadgob. At the time of data extraction (5 June 2021), the Government led in the number of tweets and number of followers/profiles followed. In contrast, Sánchez led in the number of followers. The five issued 1018 posts during the study period, more than half (53.2%) of the sample (see Table 3).

With regard to the type of messages, 91.8% of the Spanish sample are tweets, while the remaining 8.3% are mentions (6.5%) and replies (1.8%). Only Darias has a majority of mentions (88.9%). As for replies, Sánchez replied to 0.8% of his posts, Illa to 1.9%, the Ministry to 5%, and the Government and Darias to none. Therefore, all of them received a low level of interaction.

**Figure 5:**
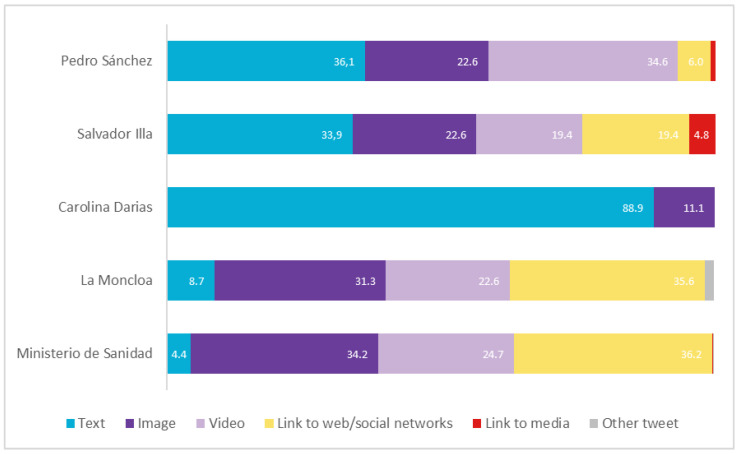
Typology of message formats in Spain in percentages. Source: Twitter and own elaboration.

In terms of format (see Figure 5), most of the posts by Sánchez (57.3%), Illa (42%), the Government (53.9%) and the Ministry (58.9%) were accompanied by images (image and video). In addition, both Illa, the Government and the Ministry included links to a large extent (24.2%, 35.6% and 36.4%, respectively).

**Figure 6:**
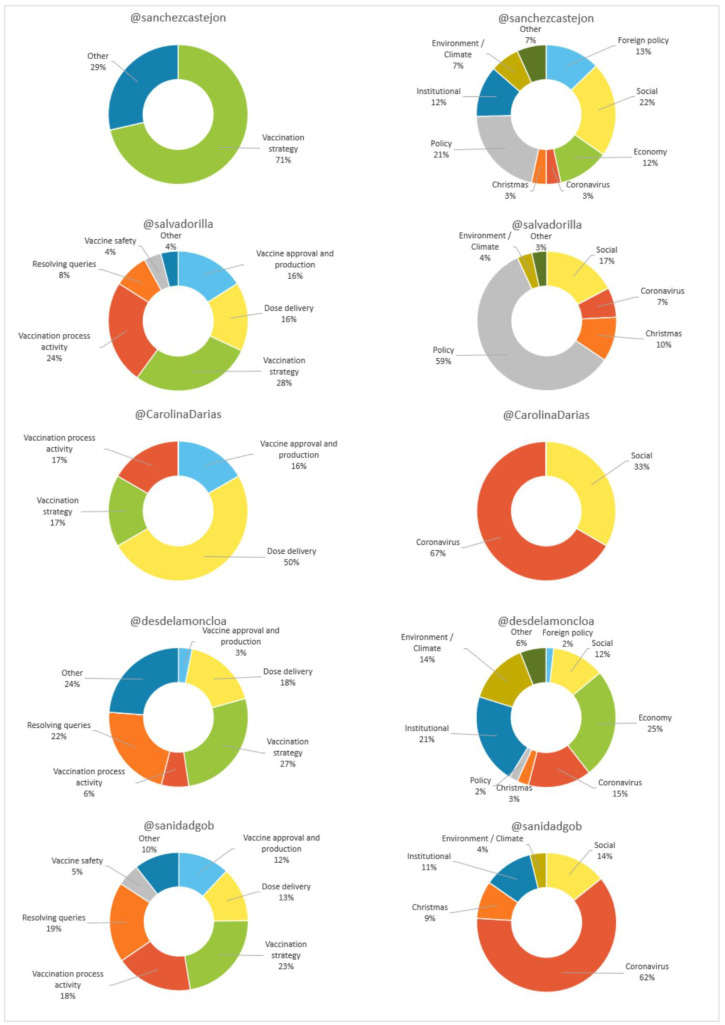
Content (vaccine related/other) of digital messages in Spain as a percentage. Source: Twitter and own elaboration.

Regarding content (see Figure 6), most of the tweets by Sánchez (94.4%), Illa (53.7%), the Government (87.7%) and the Ministry (57.9%) did not refer to the vaccine. In contrast, 66.7% of Darias’ tweets talked about it. The results also show that, in the topics related to the vaccine, “vaccination strategy” predominates in the first four profiles (71%, 28%, 27% and 23%, respectively), while in Darias’ profile “delivery of doses” predominates (50%).

In the messages that differ from the vaccine issued by Sánchez, “social” (22%) is established as the main theme; in those of Illa, issues related to “politics” (59%); and in those of La Moncloa, those related to the “economy” (25%). However, “coronavirus” dominates those published by Darias and the Ministry (67% and 62%, respectively).

**Figure 7:**
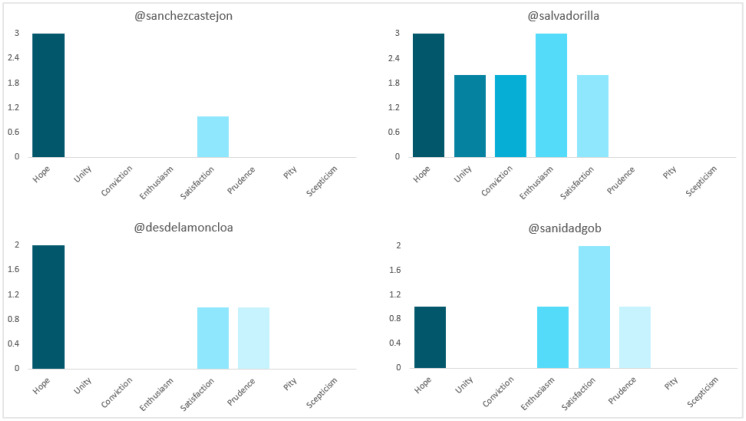
Sentiment of the tweets of Spanish politicians and institutions. Source: Twitter and own elaboration.

In terms of polarity (see Figure 7), Sánchez’s and Illa’s posts are almost evenly balanced between “positive” and “neutral” (57.1% and 42.9%; 48% and 52%, respectively). The sentiment to which the former most appeals is “hope” (75%), while the latter to “hope” (25%) and “enthusiasm” (25%). All of Darias’ (100%) are “neutral”. Additionally, the distribution for the Government and the Ministry is very similar, with a majority of “neutral” (93.7% and 96.2%, respectively). In the case of La Moncloa “hope” is the most used (50%), while in the case of the Ministry it is “satisfaction” (40%).

**Figure 8:**
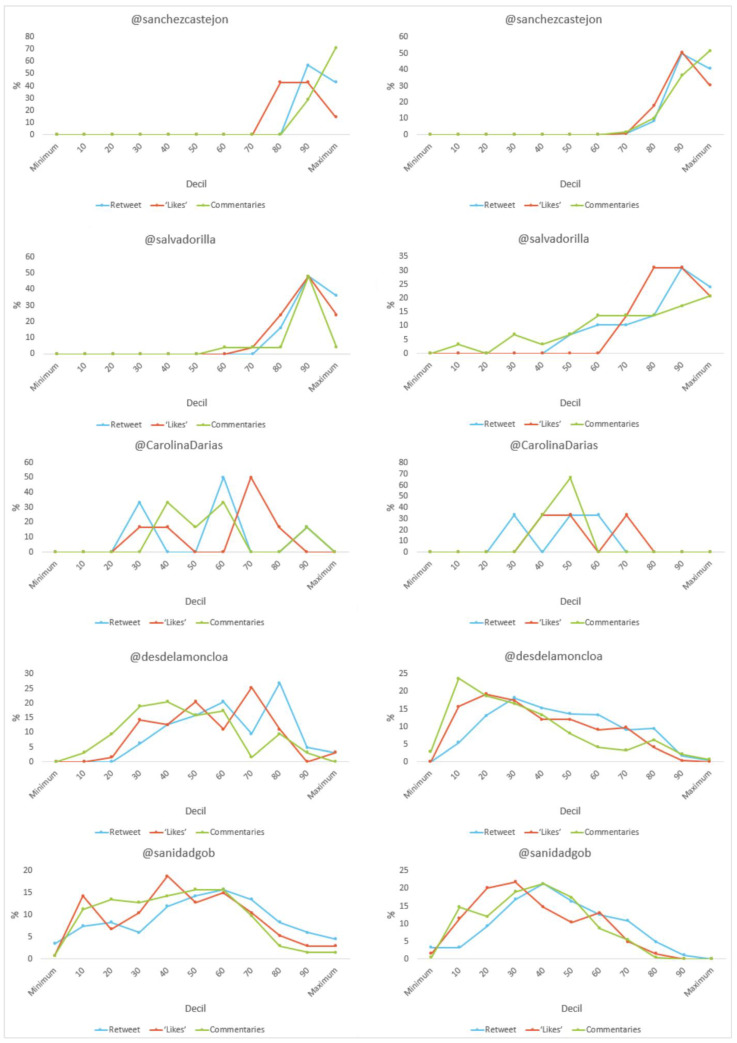
Engagement as a percentage of tweet content (total/vaccine-related) by decile in Spain. Source: Twitter and own elaboration.

In terms of the level of engagement (see Figure 8), it is worth noting that all (100%) of Sanchez’s tweets about vaccination exceed 100 “retweets”, “likes” and “comments”, while those that differ from vaccination only achieve these figures in terms of “likes”. For the latter, the majority of Illa’s publications (20.7%) are at the top in terms of the number of “comments”. On the other hand, all (100%) of Darias’ posts do not exceed 100 “retweets”. The Ministry is the only one that has posts on vaccine-related and non-vaccine related issues without sharing, liking or commenting.

### 3.3. Case Study: France

The President of the Republic, the Minister of Health, the Ministry of Health and the Government use Twitter through the accounts @EmmanuelMacron, @olivierveran, @Elysee and @Sante_Gouv. At the time of data extraction (5 June 2021), the latter led in number of followers and in followers/profiles followed. In contrast, Élysée led in the number of tweets. All four issued 382 posts during the study period, 20% of the sample (see Table 4).

With respect to the type of messages, 91.4% are tweets while the remaining 8.6% are mentions (6.8%) and replies (1.8%). Only the Ministry did not have any mentions. As for replies, Macron replied to 2.7% of his posts, the Ministry to 2.9% and the Government and Véran to none. Consequently, they all have a low level of interaction.

**Figure 9:**
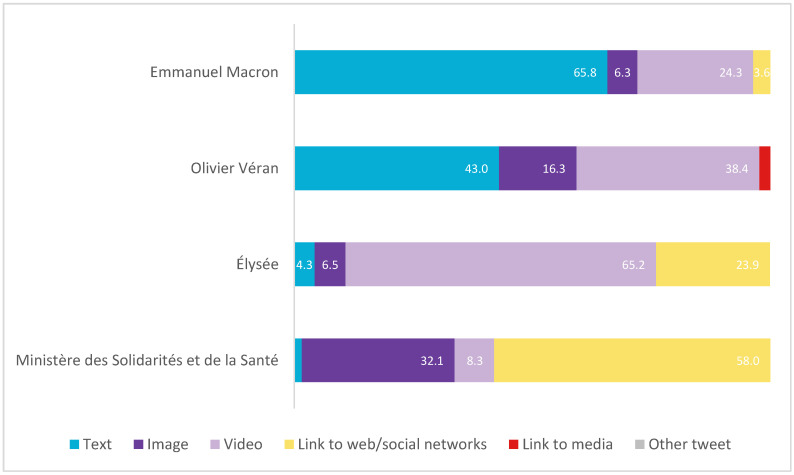
Typology of message formats in France in percentages. Source: Twitter and own elaboration.

In terms of format (see Figure 9), Véran and Élysée accompany most of their posts with images (image and video) (54.7% and 71.7%, respectively), while Macron makes greater use of text-only posts (65.8%). In the case of the Ministry, more than half (58%) include a link.

**Figure 10:**
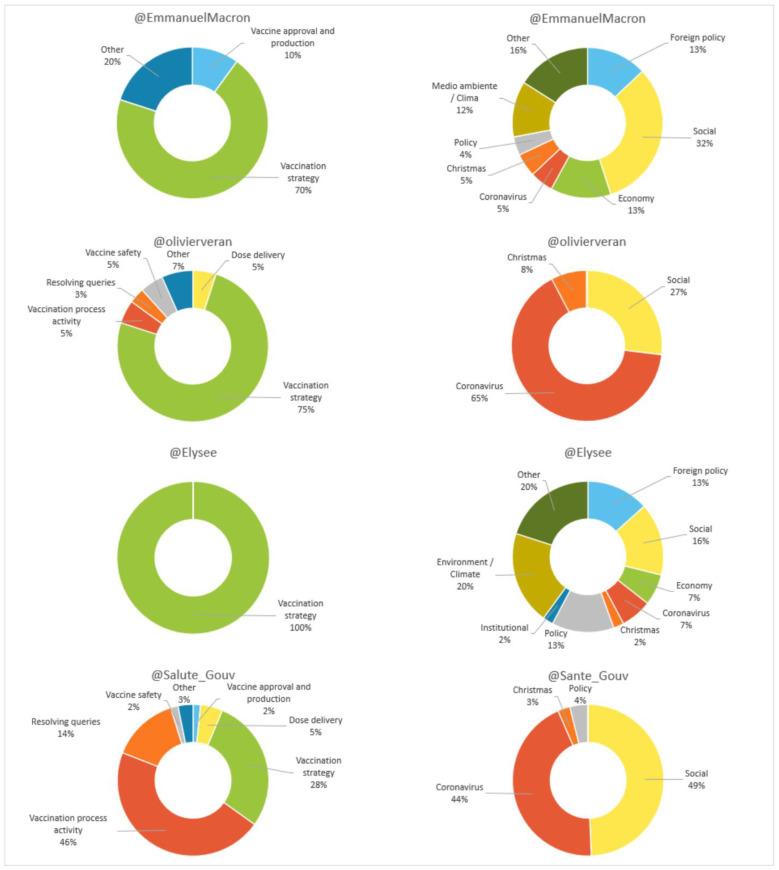
Content (vaccine related/other) of digital messages in France as a percentage. Source: Twitter and own elaboration.

In terms of content (see Figure 10), the majority of posts by Macron (90.9%), Élysée (97.8%) and the Ministry (55%) do not refer to the vaccine. Véran is the exception, with 69.8% talking about it. Our results also show that, when it comes to vaccine-related topics, “vaccination strategy” is predominant for Macron (70%), Véran (75%) and the government (100%). On the other hand, “activity of the vaccination process” is predominant for the Ministry (46%).

In the messages that differ from the vaccine issued by Macron and the Ministry, “social” (32%) is the most frequent theme (32% and 49%, respectively). In the case of Véran “coronavirus” prevails (65%), and in the case of Élysée it is “environment/climate” and “other” issues, with 20% each.

**Figure 11:**
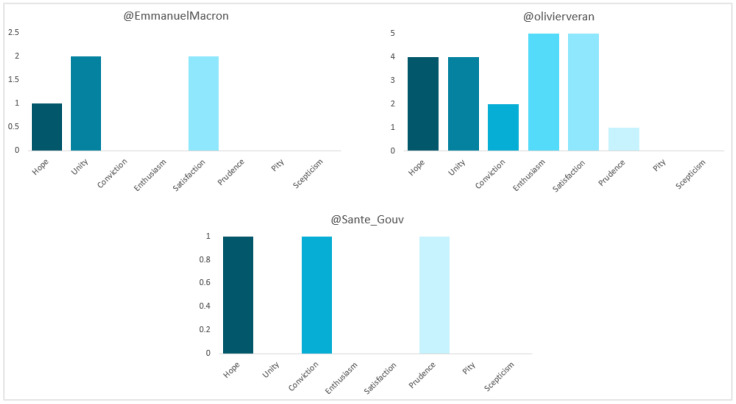
Sentiment of the tweets of French politicians and institutions. Source: Twitter and own elaboration.

In terms of polarity (see Figure 11), the majority of Véran’s and the Ministry’s publications are “neutral” (65% and 95.2%, respectively). The sentiments most appealed to by the former are “enthusiasm” and “satisfaction”, with 23.8% each, while the latter appeal to “hope”, “conviction” and “prudence”, with 33.3% each. For Macron, 50% are “neutral” and 50% “positive”, with “unity” and “satisfaction” the most popular, at 40% each. The whole (100%) of the government’s, meanwhile, are “neutral”.

**Figure 12:**
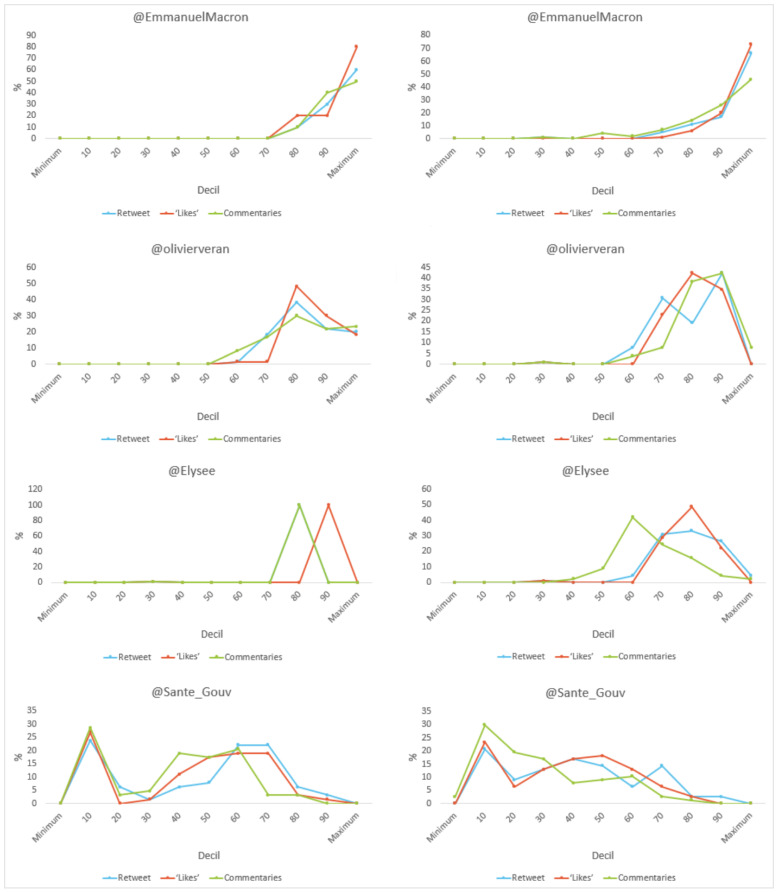
Engagement as a percentage of tweet content (total/vaccine-related) by decile in France. Source: Twitter and own elaboration.

With regard to the level of engagement (see Figure 12), it should be noted that all (100%) of the tweets by Macron and the government regarding vaccination exceeded a hundred units in terms of “retweets”, “likes” and “comments”. For content that differed from it, all (100%) of Véran’s exceeded 100 “likes”. No message about the vaccine went without interaction, while 2.6% of the Ministry’s differing messages did not receive any comments.

### 3.4. Case Study: Italy

The then-Prime Minister, Conte, the Minister of Health, the Ministry of Health and the Government used Twitter with the accounts @GiuseppeConteIT, @robersperanza, @Palazzo_Chigi and @MinisteroSalute. At the time of data extraction (5 June 2021), Conte led in number of followers and followers/profiles followed. In contrast, Palazzo Chigi led in number of tweets. All three issued 158 posts during the study period, 8.3% of the sample (see Table 5).

**Figure 13:**
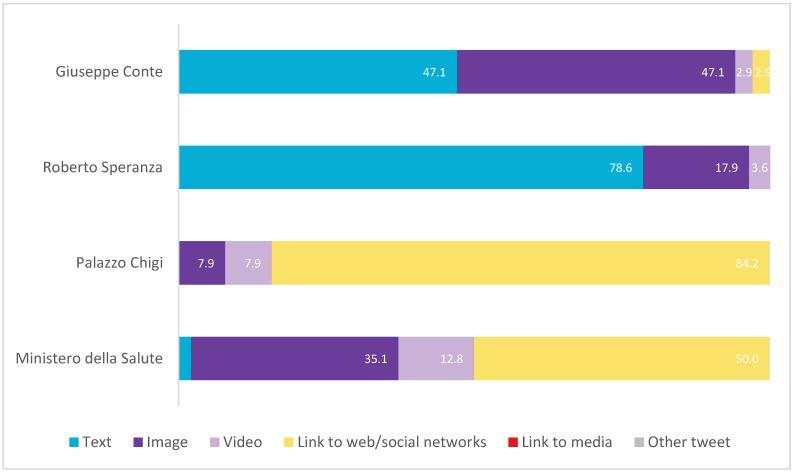
Typology of message formats in Italy in percentages. Source: Twitter and own elaboration.

With regard to the type of messages (see Figure 13), 98.7% of the total messages issued by the four accounts were tweets, while the remaining 1.3% were mentions. Only the Government and the Ministry had mentions (2.8% and 1.7%, respectively). Moreover, none of them replied to their posts. Thus, all of them had a low level of interaction.

In terms of format, most of the Ministry’s (50%) and the Government’s (84.2%) posts contain links. In contrast, Speranza does not include links. Likewise, half (50%) of Conte’s publications are accompanied by images (image and video).

**Figure 14:**
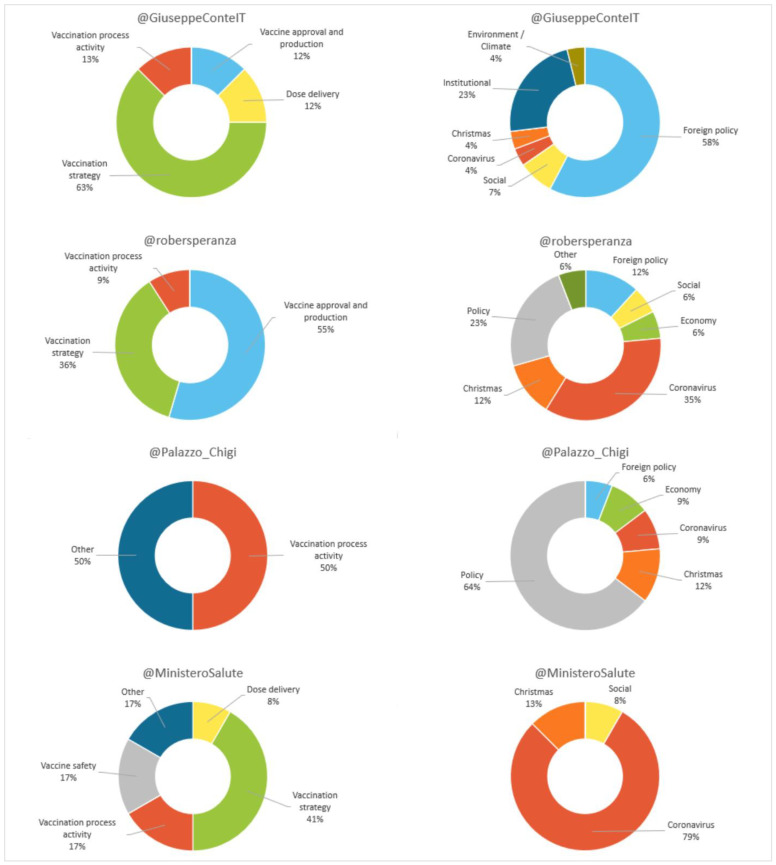
Content (vaccine related/other) of digital messages in Italy as a percentage. Source: Twitter and own elaboration.

Regarding content (see Figure 14), most of the tweets of Conte (76.5%), Speranza (60.7%), Palazzo Chigi (94.4%) and the Ministry (80%) do not talk about the vaccine. As can be seen in Figure 14, the results show that, on issues related to the vaccine, “vaccination strategy” predominates for Conte (63%) and the Ministry (41%). For Speranza more than half (55%) refer to “vaccine approval and production”, and for the Government one half (50%) to “activity of the vaccination process” and the remaining half (50%) to “other”.

In the non-vaccine messages issued by Conte, “foreign policy” (58%) is established as the main theme, and in the government’s, “policy” issues (64%). However, “coronavirus” dominates those of Speranza (35%) and the Ministry (79%).

**Figure 15:**
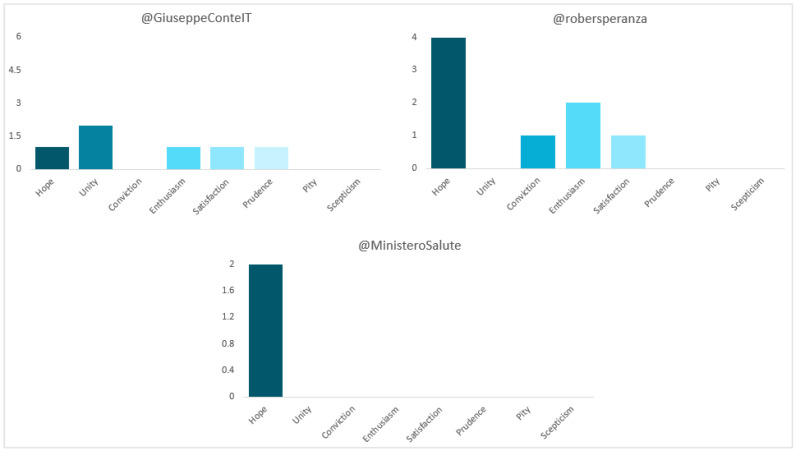
Sentiment of Italian politicians’ and institutions’ tweets. Source: Twitter and own elaboration.

In terms of polarity (see Figure 15), most of Conte’s and Speranza’s publications are “positive” (75% and 72.7%, respectively) and those of the Ministry “neutral” (83.3%). The sentiment to which the former most appeals is “unity” (25%), while the latter appeals to “hope” (50%). All (100%) of the latter corresponds to “hope”. In the case of Palazzo Chigi, all the messages (100%) are “neutral”.

**Figure 16:**
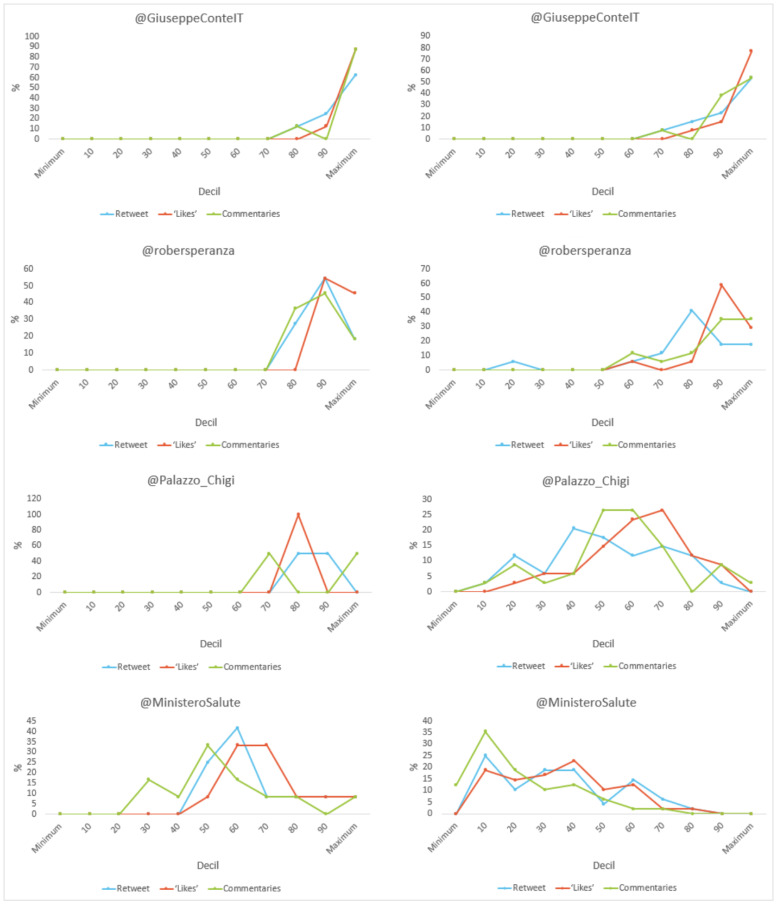
Engagement as a percentage of tweet content (total/vaccine-related) by decile in Italy. Source: Twitter and own elaboration.

With regard to the level of engagement (see Figure 16), the most relevant data show that all (100%) of the tweets about Conte and Speranza’s vaccine exceeded 100 “retweets”, “likes” and “comments”. Likewise, half (50%) of the Ministry’s publications were at the top in number of “likes”. No post about the vaccine went without interaction, while 12.5% of the Ministry’s posts that differ from the vaccine received no comments.

## 4. Discussion and Conclusions

The results of our research led to five conclusions about political and institutional communication on Twitter in Germany, Spain, France and Italy during the COVID-19 vaccination campaign that allowed us to verify or refute the research hypotheses.

**H1.** stated that, in the digital interactions of politicians and institutions with citizens, uni-directionality prevails over bi-directionality, and therefore they consolidated the use of Twitter as part of their communication strategy.

In line with previous research [13,14], politicians and institutions do not encourage dialogue with citizens and, therefore, there is a clear lack of use of the tool as a two-way communication channel. The results obtained validate this and show that, regardless of the volume of followers, followed profiles and tweets, the four cases hardly use the possibilities of mentions and replies (4.8% Germany, 8.3% Spain, 8.6% France and 1.3% Italy).

This is relevant because, despite being present on the social network, they are still anchored in the mass communication model of web 1.0 [15]. They use it only to report their actions, and tend to self-refer to it. Indeed, they give it the same function as the traditional media, ignoring its peculiarities. Thus, there is a lack of interest in maintaining an active dialogue with users (as, at a European institutional level, Papagianneas [73] or Tuñón and Carral [19] have already demonstrated), who find it impossible to debate with them and, therefore, do not feel listened to. This leads us to believe that there is still a lack of knowledge/interest in the conversational potential of social media to engage with the citizenry.

Moreover, on the basis of the results obtained the second part of our hypothesis can be also verified, albeit with certain nuances. In the German case, Chancellor Merkel and the government do not have an official profile. Moreover, except in the case of Spain, the average number of posts published is relatively low (7.1 tweets/day in Germany, 20.36 in Spain, 7.64 in France and 3.6 in Italy). Therefore, in general, they do not use the tool actively [16]. Germany, Spain and France make use of images and video (38.8%, 55.1% and 44%, respectively), while Italy uses links to expand information (41.2%). The latter is in line with previous research, which has shown that multimedia content and links are the main discursive resources used on Twitter [19].

**H2.** stated that the main content of the messages focuses on issues related to the political and institutional agenda, leaving vaccine-related issues in the background.

According to the results of our content analysis, this is valid for all our case studies, except for the German case study, which is the exception to the trend. This means that tweets from Spain, France and Italy focused mostly on non-vaccine issues (77%, 64.9% and 79.1%, respectively), which was not the case for Germany (52.7%). The data collected show that non-vaccine content focuses on “coronavirus” for Germany (54.8%), Spain (23.9%) and Italy (38.4%) and on “social” for France (33.9%).

This is in contrast to previous research [74,75,76], in which issues shaping the public agenda were relegated to minor topics within the political and institutional agenda. In our view, the divergence could be due to the specific context of the pandemic, which has been motivated by social, health and economic aspects that are relatively accepted or obvious and less susceptible to politicization (at least at the initial stage of the vaccination process).

**H3.** argued that the messages published by the European institutions and leading European officials do not present an accentuated positive polarity of a hopeful nature.

According to the results of our sentiment analysis we can refute this hypothesis, as the majority of publications by politicians and institutions in Germany (76.5%), Spain (89.3%), France (78.4%) and Italy (51.5%) had a neutral polarity. It is true, however, that within the positive messages hope was the main sentiment for Germany (42.9%), Spain (36%) and Italy (43.8%).

This is significant, as discourses that appeal to emotions can influence citizens’ decisions [77]. In our view, the opportunity to convey to users a hopeful/positive feeling about vaccination that would have contributed, from institutional profiles, to address vaccine hesitancy is missed. In fact, neither politicians nor national institutions focused their discourse on calls to action addressed to their audiences, nor were they challenged towards a pro-vaccination citizen mobilization. The main attributed function detected was related to distributing general information, in an aseptic manner, about the vaccination strategy and its implementation. 

Therefore, despite recent studies such as the one carried out by García and Rivas de Roca [74] on the 2019 European Parliament elections, which suggest that the EU planned political communication strategies on Twitter with the aim of overcoming its structural problems, the main European countries reproduce the obvious shortcomings of the digital communication strategies implemented by the EU when it comes to effectively involving and mobilizing European citizens. Indeed, they prioritize the use of Twitter and digital narratives for information purposes without taking into account the decisive potential of this social network for the creation of interpretative frameworks on a given issue, as already addressed by Caiani and Guerra [78], Papagianneas [73], and Bouza and Tuñón [25].

**H4.** stated that the contents of publications related to the vaccine tend to generate more engagement than those that differ from the vaccine.

Even taking into account that the level of commitment of politicians to the vaccine is difficult to confirm because it is considered too subjective a value, even for qualitative analysis, in view of our results this hypothesis is (somewhat) validated. This hypothesis is fully reflected in the Italian case, insofar as all three indicators are positive (51.9% retweets, 77.2% ‘likes’ and 46.3% comments). For Germany and Spain, two of the three indicators are positive (45% ‘likes’, 34.8% comments and 34.2% retweets, 49.6% ‘likes’, respectively). However, if we look at the total, all three states generate more engagement in relation to vaccination (175.4, 82.4 and 103.8, respectively). The exception is France, with all positive indicators for those that differ from the vaccine (57.6% retweets, 75.7% ‘likes’ and 48.4% comments).

On the one hand, this reinforces the studies published by authors such as Fazekas et al. [79]. On the other hand, this diverges from other similar works [80] in which informative content obtained lower engagement levels, while those whose purpose was to confront or criticise the adversary acquired higher values, or from Bouza and Tuñón [25], who argued that the profusion of excessively descriptive or institutional hashtags undermines the effective institutional intervention in the framing process on European affairs.

The testing of the set of hypotheses has also been subject to certain limitations, especially those already mentioned in the methodological section related to the detection and recording of the stakeholders involved in the issuing of the publications (which have been solved by establishing alternative indicators capable of duly quantifying this factor). In particular, the main handicap lay in the tool (Twitter API researcher) for the extraction of Twitter messages. The impossibility of its use limited our work fundamentally in the substitution of retweets for tweets cited or mentions, because although they are different concepts they are homologous and of similar usefulness in the framework of the hypotheses that pointed to the uni-directionality and bi-directionality of the messages. 

Likewise, this research has been carried out with the aim of analysing the digital communication strategies on Twitter implemented in relation to a campaign that was still underway at the end of the study’s data collection, the results of which could be affected and their validity limited by strategic modifications that may occur after that point in time. Nevertheless, it is worth noting that during the process of sample delimitation (in the first pre-analysis phase) it was possible to observe superficially how subsequent publication trends maintained the patterns found during the period finally chosen, with no major events of great resonance that altered the strategic communication of the COVID-19 vaccination campaign.

In addition, the sample selection decision has also determined the level of depth of the analysis carried out. We could have chosen more accounts in order to obtain a more representative, but perhaps less homogeneous, sample. Or, on the contrary, we could have opted for an even more homogeneous and smaller number of accounts, which would have reduced the representativeness of the sample but would have allowed for a more in-depth analysis, especially as regards the sentiment analysis part. In this sense, variables related to emotions and feelings could have been disaggregated, since they are an important part of institutional political strategies. This could undoubtedly enrich the discussion about the polarity or bias in the messages (positive/negative/neutral) or the analysis of feelings (prudence, hope, etc.), which were less interpreted, it being important to know the strategies of politicians and the institutions.

Finally, future research could certainly complement this case study by carrying out a confirmatory analysis of the findings, through: (a) extrapolation of the study to other social media such as Facebook or Instagram, the latter having been more prominent in political and institutional communication for some time now; (b) extrapolation of the analysis and comparison with that of the EU main institutions (Parliament, Commission and Council); (c) application of the analysis to later stages of the vaccination process in order to test whether the development and evolution of the vaccination process changes the digital communication of European governments and representatives on Twitter; (d) or observation of the explanatory motivations (conscious or unconscious) for the failure to take advantage of Twitter’s conversational potential in the framework of political and institutional campaigns such as that of COVID-19 vaccination.

## Figures and Tables

**Table 1:** Political and institutional profiles and number of tweets analysed.

Name/Institution	Account	Sector/Function	Country	Analysed Tweets
Steffen Seibert	@RegSprecher	Government Spokesperson	Germany	67
Jens Spahn	@jensspahn	Minister of Health		41
BMG	@BMG_Bund	Ministry of Health		247
Pedro Sánchez	@sanchezcastejon	Prime Minister	Spain	125
Salvador Illa	@salvadorilla	Minister of Health		54
Carolina Darias	@CarolinaDarias	Minister of Health		9
La Moncloa	@desdelamoncloa	Government		514
Ministerio de Sanidad	@sanidadgob	Ministry of Health		316
Emmanuel Macron	@EmmanuelMacron	Prime Minister	France	110
Olivier Véran	@olivierveran	Minister of Health		86
Élysée	@Elysee	Government		46
Ministère des Solidarités et de la Santé	@Sante_Gouv	Ministry of Health		140
Giuseppe Conte	@GiuseppeConteIT	Prime Minister	Italy	34
Roberto Speranza	@robersperanza	Minister of Health		28
Palazzo Chigi	@Palazzo_Chigi	Government		36
Ministero della Salute	@MinisteroSalute	Ministry of Health		60

Source: Twitter and own elaboration.

**Table 2:** Data on German political and institutional profiles.

Name/Institution	Tweets	Following	Followers	Followers/Following	Tweets/Day
Steffen Seibert	13.800	154	1000 K	6.493	1, 34
Jens Spahn	12.000	648	254, 5 K	392	0, 82
BMG	11.000	995	276, 2 K	277	4, 94

Source: Twitter and own elaboration. Data as of 5 June 2021.

**Table 3:** Data on Spanish political and institutional profiles.

Name/Institution	Tweets	Following	Followers	Followers/Following	Tweets/Day
Pedro Sánchez	29.400	6.032	1.500 K	248	2, 50
Salvador Illa	3.875	473	103,4 K	218	1, 46
Carolina Darias	14.700	1.748	271 K	15	0, 69
La Moncloa	46.300	199	756,8 K	3.803	10, 28
Ministerio de Sanidad	18.800	691	651 K	942	6, 32

Source: Twitter and own elaboration. Data as of 5 June 2021.

**Table 4:** French political and institutional profile data.

Name/Institution	Tweets	Following	Followers	Followers/Following	Tweets/Day
Emmanuel Macron	10.200	722	7.000 K	9.695	2, 20
Olivier Véran	8.740	463	329 K	710	1, 72
Élysée	24.100	302	2.600 K	8.609	0, 92
Ministère des Solidarités et de la Santé	16.100	652	284, 2 K	435	2, 80

Source: Twitter and own elaboration. Data as of 5 June 2021.

**Table 5:** Data on Italian politico-institutional profiles.

Name/Institution	Tweets	Following	Followers	Followers/Following	Tweets/Day
Giuseppe Conte	1.460	135	1.000 K	7.407	0, 68
Roberto Speranza	3.739	2.991	153, 4 K	51	0, 56
Palazzo Chigi	7.012	1.814	804, 6 K	443	0, 72
Ministero della Salute	4.536	225	259, 3 K	1.152	1, 20

Source: Twitter and own elaboration. Data as of 5 June 2021.

## Data Availability

The database of this research is public and was extracted from the referred Twitter accounts.

## Appendix A

**Table A1:** CODING PROTOCOL I.

Content		
Variable	Category of Analysis	
Type	1. Tweet	
	2. Mention	
	3. Response/reply	
Format	1. Text	
	2. Image (photograph, infographics and animated GIFs)	
	3. Video	
	4. Link to external website/other networks	
	5. Link to media	
	6. Other tweet	
Content	0. No mention of the vaccine	1. Foreign policy (Brexit, China, USA, etc.)
		2. Social (education, feminism, health, etc.)
		3. Economy (agriculture, employment, tourism, etc.)
		4. Coronavirus
		5. Christmas
		6. Policy
		7. Institutional (agenda events, visits to venues)
		8. Environment/climate
		9. Other (science, culture, sport, etc.)
	1. Talk about the vaccine	1. Vaccine approval and production
		2. Delivery of doses
		3. Vaccination strategy
		4. Vaccination process activity
		5. Resolution of doubts
		6. Vaccine safety
		7. Other
**Sentiment**		
**Variable**	**Category of analysis**	
Polarity	1. Positive	1. Hope
		2. Unity
		3. Conviction
		4. Enthusiasm
		5. Satisfaction
		6. Prudence
	2. Negative	1. Pity
		2. Scepticism
	3. Neutral	

## Appendix B

**Table A2:** CODING PROTOCOL II.

		Retweet	‘Likes’	Commentaries
N		1.913	1.913	1.913
Minimum		0	0	0
P E R C E N T I L	10	10	25	3
	20	18	36	6
	30	25	49	10
	40	34	68	16
	50	47	103	27
	60	72	169	48
	70	116	329	97
	80	196	742	207
	90	420	1.891	492
Maximum		15.700	86.400	8.800
